# Sialidases from gut bacteria: a mini-review

**DOI:** 10.1042/BST20150226

**Published:** 2016-02-09

**Authors:** Nathalie Juge, Louise Tailford, C David Owen

**Affiliations:** * Institute of Food Research, The Gut Health and Food Safety Institute Strategic Programme, Norwich Research Park, Norwich NR4 7UA, U.K.; † Biomolecular Sciences Building, University of St Andrews, St Andrews, Fife, KY16 9ST, UK

**Keywords:** sialidase, gut bacteria, gut microbiota, glycoside hydrolase, sialic acid, mucin degradation

## Abstract

Sialidases are a large group of enzymes, the majority of which catalyses the cleavage of terminal sialic acids from complex carbohydrates on glycoproteins or glycolipids. In the gastrointestinal (GI) tract, sialic acid residues are mostly found in terminal location of mucins via α2-3/6 glycosidic linkages. Many enteric commensal and pathogenic bacteria can utilize sialic acids as a nutrient source, but not all express the sialidases that are required to release free sialic acid. Sialidases encoded by gut bacteria vary in terms of their substrate specificity and their enzymatic reaction. Most are hydrolytic sialidases, which release free sialic acid from sialylated substrates. However, there are also examples with transglycosylation activities. Recently, a third class of sialidases, intramolecular *trans*-sialidase (IT-sialidase), has been discovered in gut microbiota, releasing (2,7-anhydro-Neu5Ac) 2,7-anydro-*N*-acetylneuraminic acid instead of sialic acid. Reaction specificity varies, with hydrolytic sialidases demonstrating broad activity against α2,3-, α2,6- and α2,8-linked substrates, whereas IT-sialidases tend to be specific for α2,3-linked substrates. In this mini-review, we summarize the current knowledge on the structural and biochemical properties of sialidases involved in the interaction between gut bacteria and epithelial surfaces.

## Sialic acid metabolism in the gut

**Figure 1:**
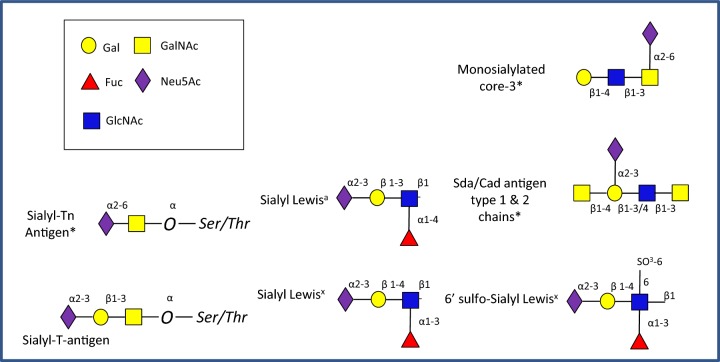
Sialylated terminal glycan structures in the gut Structures shown are representative and not exhaustive. These glycans are appended to the core mucin structures. In mice the fucose (Fuc) residues are more commonly linked to Gal rather GlcNAc, *structures common in human and rare in mice.

In the gastrointestinal (GI) tract, sialic acid [*N*-acetylneuraminic acid (Neu5Ac)] is commonly found in terminal location of mucins [1,2]. Mucins are large glycoproteins, which can be broadly grouped as membrane-bound or secreted [3]. Membrane-bound mucins are essential contributors of the glycocalyx of mucosal surfaces where they play important biological roles in cell interactions and signalling [4]. Secreted mucins are the main structural components of the mucus gel covering the epithelium and essential to the maintenance of a homoeostatic relationship with our gut microbiota [1]. Mucins are characterized by a proline–threonine–serine (PTS) domain which is the site of extensive O-glycosylation with carbohydrates accounting for up to 80% of the total mucin mass. The synthesis of mucin oligosaccharides starts with the transfer of *N*-acetyl-galactosamine (GalNAc) to serine and threonine residues of the mucin backbone to form mucin O-glycan core structures [5]. These core structures can be further elongated with galactose (Gal), *N*-acetyl-glucosamine (GlcNAc), GalNAc and frequently modified by terminal fucose or sialic acid residues via α1-2/3/4 and α2-3/6 linkages, respectively (Figure 1). The proportion of the major mucin glycan epitopes, sialic acid and fucose, varies along the GI tract with a decreasing gradient of fucose and an increasing gradient of sialic acid from the ileum to the rectum in humans [6] and a reverse gradient in mice [7]. The Neu5Ac α2-6 *N*-acetylgalactosaminitol epitopes and Sda/Cad antigens found in humans [6,8] are absent or rare in mice where as the Neu5Ac–GlcNAc epitope and disialylated epitopes are more common along the murine GI tract [7].

The GI tract is heavily colonized with bacteria. Most species belong to the phyla Firmicutes, Bacteroidetes, Actinobacteria, Proteobacteria and Verrucomicrobia. The microbiota composition varies longitudinally along the GI tract but also transversally from the mucosa to the lumen [9,10]. The terminal mucin O-glycans have been proposed to serve as metabolic substrates, providing a nutritional advantage to bacteria which have adapted to the GI mucosal environment [11,12]. The release of sialic acid from non-reducing ends by sialidases is an initial step in the sequential degradation of mucins, since the terminal location of sialic acid residues in the mucin oligosaccharide chains may prevent the action of other glycoside hydrolases (GHs). In bacteria, the genes involved in sialic acid metabolism are usually found clustered together forming what is denominated as a Nan cluster. The canonical *nanA/K/E* cluster was first described in *Escherichia coli* [13] and an alternative pathway defined by the *nanLET* cluster was later discovered in *Bacteroides fragilis* [14]. The majority of the bacteria that harbour a Nan cluster colonize mucus regions of the human body, such as the gut where sialic acid is highly abundant and can serve as a source of energy, carbon and nitrogen [15]. As described below, a number of gut bacteria employ sialidases in the release of host sialic acids, including multiple species of *Clostridia* [16], *Bacteroides* [17], certain subspecies/serovars/strains of *Bifidobacterium longum* [18], *Vibrio cholerae* [19], *Ruminococcus gnavus* and *Akkermansia muciniphila* [20]. However, some bacteria appear to have incomplete packages of enzymes for utilizing host sialic acids. For example, *Bacteroides thetaiotaomicron* VPI-5482 encodes a sialidase and can release free sialic acid, but lacks the Nan operon required to consume the liberated monosaccharide and does not appear capable of consuming free sialic acid [21]. On the other hand *Clostridium difficile* strain 630 encodes the Nan operon but lacks the sialidase [22] and thus relies on other sialidase-producing organisms to acquire this potential nutrient source from the mucosal environment [23,24]. In contrast, some bacteria appear to possess the complete pathway of sialic acid catabolism including a predicted sialidase gene e.g. *B. fragilis* strains [14,25]. A recent study reported that mice monoassociated with *B. thetaiotaomicron* exhibited a significantly higher concentration of free Neu5Ac compared with germ-free mice, consistent with the ability of *B. thetaiotaomicron* to liberate but not consume the monosaccharide, whereas colonization of mice with *B. fragilis*, which is able to catabolize Neu5Ac, did not result in increased free sialic acid [24].

In contrast with gut commensals, which appear to use sialidases primarily for nutrient acquisition, some pathogens of the GI tract such as *V. cholerae* or *Clostridium perfringens* strains also use sialidases to decrypt adhesin or toxin-binding sites [26]. All toxigenic strains of *V. cholerae* have a sialidase encoded within a pathogenicity island in their genomes [27]. However it is also worth noting that, within particular pathogenic or commensal species, the presence or absence of sialidase-encoding genes in bacterial genomes is often strain-specific. For example *R. gnavus* ATCC 29149 but not E1 expresses a sialidase [20,28], several strains of *E. coli*, e.g. enteropathogenic *E. coli* O127 strain (EPEC) [29] or probiotic strain Nissle 1917 [30] possess a sialidase-encoding gene whereas commensal *E. coli* strains such as *E. coli* strain EHV2 lack a sialidase [31]. However *E. coli* sialidases remain to be biochemically characterized. Similarly, not all *Salmonella enterica* strains encode a putative sialidase and only one sialidase has been functionally-characterized from *S. enterica* serovar typhimurium although it appears to have been acquired by horizontal transfer [32,33].

Sialic acid catabolism in the gut is important as increased free sialic acid levels in the intestinal mucosal compartment, e.g. post-antibiotic treatment, will favour outgrowth of some bacterial pathogenic strains of *S. Typhimurium* and *C. difficile* [24] or the outgrowth of *E. coli* during inflammation [31]. Such cross-feeding activity has also been reported between commensal bacteria, e.g. *Bifidobacterium breve* UCC2003 (containing a functional Nan cluster for sialic utilization) can utilize sialic acid released by the sialidase activity of *Bifidobacterium bifidum* PRL201048 [34]. The gut symbiont, *R. gnavus* ATCC 29149, is different from the above as it possesses the complete Nan cluster and an intramolecular *trans*-sialidase (IT-sialidase), thus producing (2,7-anhydro-Neu5Ac) 2,7-anydro-*N*-acetylneuraminic acid instead of free Neu5Ac, suggesting a novel mechanism of adaptation to the mucosal environment [20]. The biological role of bacterial sialidases produced by human gut commensal and pathogenic bacteria has been reviewed previously [15,26,35]. Here we focus on the structural and biochemical properties of characterized sialidases involved in the interaction between gut bacteria and epithelial surfaces.

## Sialidases from gut bacteria: structure and mechanism of action

### General features

**Figure 2:**
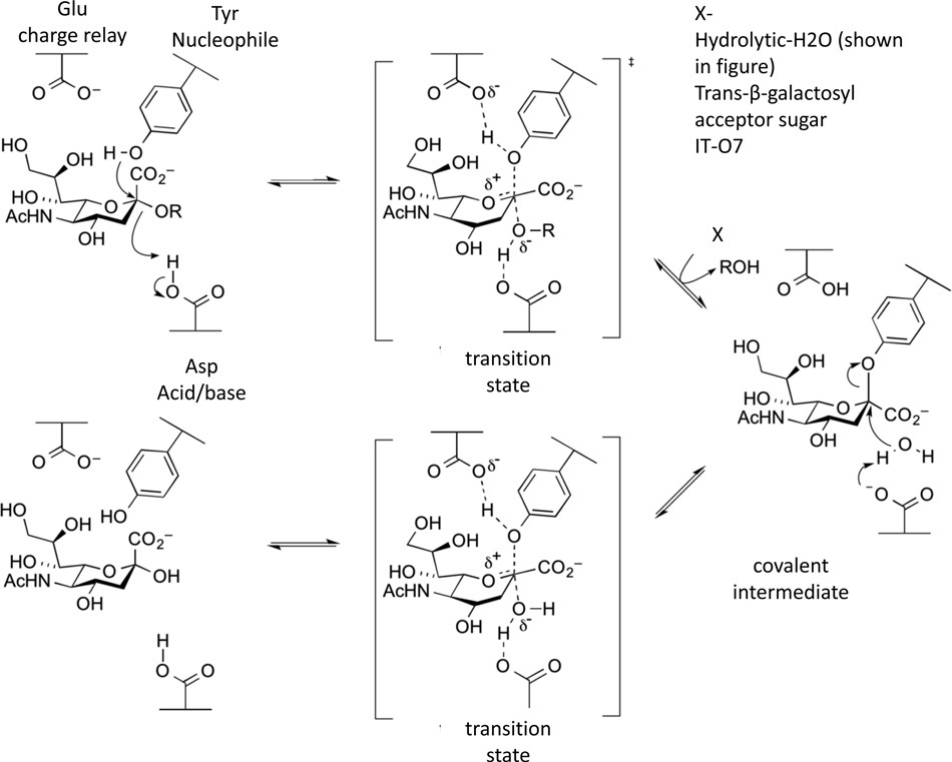
Mechanism of action hydrolytic/*trans*/IT-sialidases sialidases act via a two-step double-displacement mechanism so that the α-configuration of the glycosidic bond is retained The glycosylation step is the same for all three classes of sialidase, but for the deglycosylation step the incoming molecule can be water, another sugar or the internal oxygen atom, as indicated. Figure adapted from www.cazypedia.org.

Sialidases (also commonly referred to as neuraminidases) are a large group of enzymes, the majority of which are exo-sialidases catalysing the cleavage of terminal sialic acids from complex carbohydrates on glycoproteins or glycolipids. Based on amino acid sequence similarities, bacterial exo-sialidases are classified in the GH family 33 (GH33) of the CAZy classification (www.cazy.org) [36]. Hydrolysis occurs via an acid/base-catalysed double-displacement mechanism involving a covalent sialyl–enzyme intermediate, resulting in overall retention of configuration at the anomeric centre [37,38] (Figure 2). Unusually the catalytic nucleophile is a tyrosine residue activated by a proximal glutamic acid, due to the charge on sialic acid itself as shown biochemically [39] and confirmed structurally [40] (Figure 2). Sialidases from the retaining sialidase families GH34 and GH83 are both restricted to viruses and examples of GH58 inverting endo-sialidases are found in some *E. coli* strains (www.cazy.org).

Based on their substrate specificity and catalytic mechanism, exo-sialidases can be separated into three classes: hydrolytic, *trans*-sialidases and IT-sialidase. Hydrolytic-sialidases cleave the glycosidic bond of terminal sialic acids and release free sialic acid, whereas *trans*-sialidases transfer the cleaved sialic acid to other glycoconjugates; according to the Enzyme Commission both classes belong to exo-α-sialidases (EC 3.2.1.18). Hydrolytic-sialidases usually have wide substrate specificity and cleave α2-3-, α2-6- and α2-8-linked terminal sialic acids. *Trans*-sialidase activity with specificity for α2-3-linked substrates was first discovered for the *Trypanosoma cruzi* sialidase TcTS [41]. *Trans*-sialidases with activity against α2-6- and α2-8-linked sialic acid substrates have been discovered in the intervening years [42,43]. The third class is the IT-sialidase (EC 4.2.2.15). Currently, the discovered and characterized IT-sialidases are strictly α2-3-linkage specific and produce 2,7-anhydro-Neu5Ac [20,44,45]. However, the substrate and linkage specificity of sialidases is often unknown due to reliance on artificial substrates such as 4-methylumbelliferyl-Neu5Ac (4MU-Neu5Ac) or 2-*O*-(*p*-Nitrophenyl)-α-D-Neu5Ac (PNP-Neu5Ac; Table 1).

**Table 1:** Characterized gut commensal and pathogenic sialidases Abbreviations: AGP- human alpha1-acid glycoprotein; BSM, bovine submaxillary mucin; GM1, monosialotetahexosylganglioside; KDN, 2-keto-3-deoxy-D-glcero-D-galactonic acid; Neu5Prop, *N*-propionylneuraminic acid; PGM, pig gastric mucin.

Bacterial species and strain	Protein name	Uniprot/Genbank	PDB	Domains	P/E*	Substrates tested	+/–^†^	References
*Akkermansia muciniphila* ATCC BAA-835/DSM 22959	Amuc_0625/ Am0707^‡^	B2UPI5		GH33	P	4MU-Neu5Ac, α2,3-, AGP, Fetuin α2,6- linkages, asialofetuin Neu5Ac-,Neu5Gc-, Neu5Prop-, KDN-	+ + – +	[20] [58]
	Amuc_1835/ Am2085‡	B2UN42		GH33	P	4MU-Neu5Ac, α2,3-, AGP, Fetuin α2,6- linkages, asialofetuin Neu5Ac-, Neu5Gc-, Neu5Prop- KDN-	+ + – + –	[20] [58]
	Amuc_0623/ Am0705^§^	B2UP13		GH33	P	Neu5Ac-, Neu5Gc- Neu5Prop-, KDN-	++ +	[58]
	Am_1547/ Am1757^§^	B2ULI1		GH33	P	Neu5Ac-, Neu5Gc-, Neu6Prop-, KDN-	+	[58]
*B. fragilis* YCH46/ TAL2480	sialidase (BF1729)	P31206		GH33	P	4MU-Neu5Ac	+	[50]
*B. fragilis* SBT3182^‡^					P	colominic acid (α2-8) α2-3 and α2-6 Neu5Ac-Lac	++ +	[51,52]
*B. fragilis* 4852^‡^					P	α2,3-, linear 2,6- and 2,8- linkages, branched sialylconjugates	++ +	[54]
						GM1 and mixed ganglosides	–	
						Mucin, fetuin, AGP and other sialylated glycoproteins β-linked sialylconjugates	+ –	
*B. fragilis* YM4000^‡^					E	4MU-Neu5Ac	+	[53]
*B. thetaiotaomicron* VPI-5482	sialidase (BtsA;BTSA;BT0455)	Q8AAK9	4BBW	GH33	P	α2,3-, 2,6- and 2,8- linked sialylconjugates fetuin, AGP, transferrin	+ +	[49]
*B. vulgatus* ATCC 8482/DSM 1447/NCTC 11154^‡^	BVU_4143	A6L7T1		GH33		4MU-Neu5Ac, PNP-Neu5Ac	+	[31]
*B. bifidum* JCM 1254	exo-α-sialidase (SiaBb2;BBP_0054)	BAK26854.1		GH33	P	4MU-Neu5Ac α2,3-, 2,6- and 2,8- linked sialylconjugates (2,3- linkages preferred), gangliosides, fetuin, PGM, hen egg yolk N-glycans also transfers Neu5Ac to 1-alkanols	+ + + +	[56]
*Cl. perfringens* A99	sialidase 1 'small'	P10481		GH33	P	4MU-Neu5Ac	+	[68]
*C. perfringens* ATCC 10543	sialidase 2 (NanH)	Q59311		GH33	P	4MU-Neu5Ac	+	[69]
*C. perfringens* ATCC 13124^║^	sialidase (CPF_0721)	Q0TT67	4L2E	CBM40, GH33	P	4MU-Neu5Ac	+	[70]
*C. perfringens* str 13^║^	exo-α-sialidase (NanI;CPSA;CPE0725)	Q8XMG4	2BF6 2VK5 2VK6 2VK7	CBM40, GH33	P	Fetuin, BSM, colominic acid, bovine brain gangliosides Can also hydrate 2-deoxy-2,3-dehydro-Neu5Ac acid to Neu5Ac	+ +	[68] [66]
*C. perfringens* str 13/ ATCC 13124^║^	exo-α-sialidase (NanJ;CPE0553	Q8XMY5	2V73[A,B]	CBM32, CBM40, GH33	P	Only the CBMs are characterized		[48]
*Clostridium tertium* ATCC 14573	sialidase (NanH;SiaH)	P77848		CBM40, GH33	P	4MU-Neu5Ac	+	[78]
*R. gnavus* ATCC 29149	*Rg*NanH^‡^	A7B557		CBM40, GH33	P	4MU-Neu5Ac, α2,3-, AGP, Fetuin α2,6-linkages, asialofetuin Releases 2,7 anhydro-Neu5Ac	+ – +	[20]
*S. typhimurium* TA262/LT2	sialidase (NanH;STSA)	P29768	1DIL 1DIM 2SIL 2SIM 3SIL	GH33	P	4MU-Neu5Ac α2-3 Neu5AcLac α2-6 Neu5AcLac, gangliosides, mucin, fetuin, colominic acid 4MU-Neu5Ac>MU-Neu5Gc	+ ++ + +	[60] [59] [63]
						Can produce Neu5Ac2en	+	[19]

*P/E refers to whether the characterization is carried out with purified (P) enzymes (including recombinant enzyme) or with bacterial extract (E).^†^This column indicates whether the enzyme is active (+) or not (–) against the substrates tested, ++ is used to denote more activity than +, where relative activity is indicated.^║^These strains are ‘flesh-eating’ strains isolated from gangrene rather than gut bacteria but are included because more biochemical data are available.^‡^Details of enzymes are not currently in CAZy ‘characterized’ page.

**Figure 3:**
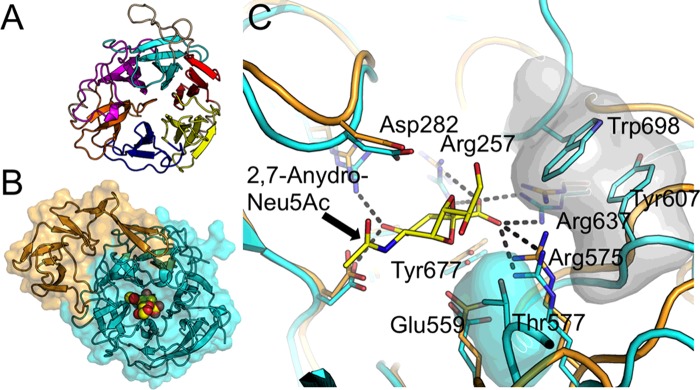
Structural features of sialidases and IT-sialidases from gut bacteria (**A**) Cartoon representation of the GH33 catalytic domain from *S. typhimurium* NanH/STSA sialidase (PDB: 1DIL). The canonical six-bladed β-propeller fold is highlighted with alternate colouring of the propeller blades. (**B**) *R. gnavus Rg*NanH IT-sialidase GH33 catalytic domain (cyan) with inserted domain (orange; PDB: 4X4A). 2,7-anhydro-Neu5Ac is shown bound into the active site. (**C**) The active site of *R. gnavus Rg*NanH (cyan) with 2,7-anhydro-Neu5Ac bound (yellow). Selected hydrogen bonds are highlighted with black dashed lines. Two characteristic features of the IT-sialidase active site are highlighted with a semi-transparent surface: the hydrophobic stack responsible for α2-3 linkage specificity (grey) and the threonine residue responsible for sterically hindering the ligand glycerol group (cyan). The *S. typhimurium* NanH/STSA active site (orange) has been superimposed, demonstrating that the majority of active site features are conserved across the hydrolytic and IT-sialidase classes. Residue numbering refers to *Rg*NanH.

The GH33 catalytic domains adopt a six-bladed β-propeller fold (Figures 3A and 3B). GH33 catalytic domains are often associated with additional domains [46] including membrane-binding domains [47] and carbohydrate-binding modules (CBMs) such as sialic acid-specific CBM40 [19,20] and broadly specific CBM32 [48] as classified in CAZy (www.cazy.org). CBMs are believed to mediate adherence of the enzyme to cognate carbohydrate substrates and enhance the hydrolase activity of the catalytic domains by increasing enzyme substrate proximity [49]. Both exo- and *trans*-sialidases share a set of active site residues and cleave the terminal α-linked sialic acid residue by the same catalytic mechanism. This conserved active site includes a glutamic acid–tyrosine charge relay with the tyrosine acting as the catalytic nucleophile [40] and an aspartate residue as the general acid/base (Figure 2). The incoming sialic acid residue is orientated in the active site via a trio of arginines, which co-ordinates the sialic acid carboxylate moiety and a hydrophobic pocket which accommodates the ligand N-acetyl group (Figure 3C). Aspartic acid-boxes are motifs commonly found at the termini of sialidase β-propeller blades, they may stabilize the protein fold by providing inter-blade contacts [50,51].

## Hydrolytic exo-sialidases

Among GI commensals, Bacteroidetes species are found at high abundance and many of them express sialidases in culture [17]. *B. thetaiotaomicron* spp. encode the sialidases required to cleave and release terminal sialic acid from the mucosal glycoconjugates, but do not encode the Nan cluster required to consume the liberated monosaccharide [21]. The purified sialidase from *B. thetaiotaomicron* VPI-5482 has been shown to hydrolyse sialylglycoconjugates including fetuin and transferrin [52] (Table 1). Presumably, the release of sialic acids allows *B. thetaiotaomicron* to access highly coveted underlying carbohydrates in the mucus. Recently, a sialidase from *Bacteroides vulgatus* BVU 4143 has been shown to be active against 4MU-Neu5Ac and PNP-Neu5Ac and inhibited by *N*-acetyl-2,3-didehydro-2-deoxyneuraminic acid (Neu5Ac2en) inhibitor [31] (Table 1). *B. fragilis* strains are among those bacteria that have been shown to possess the complete pathway of sialic acid catabolism including the sialidase. Several sialidases from different *B. fragilis* strains have been characterized, showing a broad specificity with some preference for the α2-8 linkage (Table 1) [53–57]. Sialidases have also been identified in the genomes of infant-derived Bifidobacteria, including two intracellular sialidases from *B. longum* subsp. *infantis* ATCC 15697 [18], two predicted extracellular exo-α-sialidases of *B. bifidum* PRL 2010 [58] and a putative sialidase from *B. breve* UCC2003 [34]. However, the only sialidase from this group of infant-associated bacteria to be functionally characterized is SiaBb2 from *B. bifidum* JCM 1254, a strain for which the genome sequence is not yet publicly available. SiaBb2 has a strong preference for α2-6 linkages and was shown to be sufficient to confer *B. longum* 105-A with the ability to degrade human milk oligosaccharides (HMOs) [59]. This sialidase can also transfer Neu5Ac to 1-alkanols at high acceptor concentrations [59] (Table 1). All four putative sialidases annotated in the genome of the mucin-degrading bacteria *A. muciniphila* ATCC BAA-835 [60] have recently been characterized [20,61]. The enzymes are active against a range of sialylated substrates with either α2-3 or α2-6 linkages (Table 1).

Among gut pathogens, NanH/STSA from *S. typhimurium* TA262/LT2 strain has been biochemically [62,63] (Table 1) and structurally [64,65] characterized (Figure 3A), revealing conservation of key catalytic residues with the GH34 viral sialidases, including the nucleophilic charge relay, the aspartic acid acid/base and the arginine triad. This enzyme shows kinetic preference for sialyl α2-3 linkages over sialyl α2-6 linkages [62] and preferentially cleaves Neu5Ac residues rather than *N*-glycolylneuraminic acid (Neu5Gc) residues [66] (Table 1). Some strains of *C. perfringens* encode multiple sialidases (Table 1) [67–74]. The evolutionary rationale for this is unclear but may be because the enzymes differ in their cellular location, properties and sensitivities to inhibitors [74]. NanI from *C. perfringens* is unusual in that it is a hydrolytic enzyme which can also hydrate the inhibitor Neu5Ac2en to Neu5Ac *in vitro* [69]. These enzymes differ from *V. cholerae* sialidases which can hydrolyse both α-2,3- and α-2,6-linked sialic acid substrates [75] and produce the Neu5Ac2en inhibitor [19] (Table 1). The active site has many features in common with other viral and bacterial sialidases but, uniquely, has an essential Ca^2+^ ion which plays a crucial structural role [19,76].

## *Trans*-sialidases and IT-sialidases

Most *trans*-sialidases have been characterized from trypanosome species [77]. *Trans*-sialidases have not been reported in the gut microbiota. However a few examples of exo*-*sialidases from gut bacteria have been reported to perform *trans*-glycosylation reactions under certain experimental conditions. These include the aforementioned SiaBb2 from B. bifidum and NanI from *C. perfringens* [69] (Table 1).

IT-sialidases are unique in that they catalyse an intramolecular reaction in which the O7-hydroxy group of the bound sialic acid glycerol group attacks the positively charged C2 atom of the oxocarbenium intermediate [44,78]. The altered reaction pathway leads to release of 2,7-anhydro-Neu5Ac instead of Neu5Ac (Figure 2). The first example of this enzyme class was described in NanL [79,80], which is purported to be from the leech *Macrobdella decora*, but may be from a bacterial source in the leech GI tract, as previously suggested [81]. Three IT-sialidases have been biochemically and structurally described: NanL [44,78], NanB from *Streptococcus pneumonia* [45] and *Rg*NanH from *R. gnavus* [20] (Figure 3B). The active site of IT-sialidases is characterized by a conserved threonine residue which sterically hinders the substrate glycerol group, forcing it into an axial position whence it can attack at the anomeric C2 carbon and form the intramolecular linkage [44,78] (Figure 3C). An additional characteristic feature of these enzymes is a hydrophobic rim close to the arginine triad, formed by tryptophan–tyrosine stack [20,44,45]. This feature provides strict specificity for α2-3-linked substrates and may also provide an important contribution to the reaction mechanism by providing a desolvated, hydrophobic environment. This allows the intramolecular reaction to proceed, as the O7 hydroxy of the glycerol group must outrun any incoming water molecules that would otherwise attack the C2 carbon and produce Neu5Ac (Figure 3C).

*Rg*NanH from *R. gnavus* ATCC 29149 is the first example of an IT-sialidase functionally characterized in gut bacteria [20,28]. The enzyme produces 2,7-anhydro-Neu5Ac with strict specificity towards α2-3 glycosidic substrate linkages. *Rg*NanH is a three-domain modular protein with an N-terminal lectin-like domain (L-domain) classified as a CBM40, a GH33 catalytic domain (N-domain) and a domain inserted into the catalytic domain (I-domain). The crystal structure of the *Rg*NanH catalytic domain has been solved and demonstrates the six-bladed β-propeller fold characteristic of sialidases [20]. A domain of unknown function protrudes from between two blades of the β-propeller (I-domain). Crystal structures in complex with 2,7-anhydro-Neu5Ac and known inhibitors of hydrolytic sialidases, allowed interrogation of the active site. Of particular importance is the conservation of the active site threonine (Thr^557^), which is proposed to sterically force the substrate glycerol group into a position from where it can attack the C2 atom [20] (Figure 3C). This residue also impacts on the response to sialidase inhibitors, as shown by poor inhibition by Neu5Ac2en and micromolar inhibition by siastatin B [20].

Bioinformatics analyses revealed that the presence of IT-sialidases is shared by other members of the gut microbiota, in particular *Blautia hansenii*, *Ruminococcus torques*, all 10 strains of *Clostridium perfringens* with available genome data, *C. sp.* 7 2 43 FAA, *C. celatum*, *C. nexile*, *C. spiroforme*, three unclassified Lachnospiraceae, more than 100 strains of *Streptococcus agalactiae* and three of the genome-sequenced publicly available *Lactobacillus salivarius* strains. The detection of IT-sialidase homologues in at least 11% of gut metagenomes of a population of diseased and healthy humans confirmed that this enzyme is widespread across gut bacteria, especially in Firmicutes. This analysis also revealed a greater abundance of IT-sialidase encoding species in patients with inflammatory bowel diseases (IBD) as compared with healthy individuals [20]. The specific niche colonization of these bacteria may reflect their adaptation to particular mucin glycosylation profiles associated with intestinal inflammation and/or infection [82,83].

## Conclusions and perspectives

Bacterial sialidases and their sialoglycan targets contribute to host–microbe interactions at the mucosal surface. An imbalance in the proportion of gut commensals able to modulate mucosal sialic acid levels or a change in host mucin sialylation is often associated with enteric infection or intestinal inflammation. Maintaining a balance in the ability of gut commensals to produce and/or consume sialic acid in the mucosal compartment is therefore essential to gut homoeostasis.

Further investigations of bacterial sialidases should clarify the type of sialylated structures that are accessible to the gut bacteria and the specificity of sialidases towards sialic acids with different modifications and in different linkages. These include gaining structural insights into the diversity of sialic acid derivatives that can be produced and/or taken up by commensal and pathogenic bacteria. Thus, for therapeutic purposes, modulation of sialidase expression might be effectively achieved by appropriate use of specific inhibitors or pro/prebiotic approaches targeting specific bacterial strains.

## Abbreviations

2,7-anydro-Neu5Ac: 2,7-anydro-*N*-acetylneuraminic acid
4MU-Neu5Ac: 2′-(4-methylumbelliferyl)-α-D-*N*-acetylneuraminic acid
CBM: carbohydrate-binding module
Gal: galactose
GalNAc: *N*-acetyl-galactosamine
GH: glycoside hydrolase
GH33: GH family 33
GI: gastrointestinal
GlcNAc: *N*-acetyl-glucosamine
IT-sialidase: intramolecular *trans*-sialidase
MU: 4-Methylumbelliferonemethylumbelliferone
Neu5Ac: *N*-acetylneuraminic acid
Neu5Ac2en: *N*-acetyl-2,3-didehydro-2-deoxyneuraminic acid
Neu5Gc: *N*-glycolylneuraminic acid
PNP-Neu5Ac: 2-*O*-(*p*-Nitrophenyl)-α-D-*N*-acetylneuraminic acid
PTS: proline-threonine-serine
